# Anaesthetic Management of Parturient with Acute Atrial Fibrillation for Emergency Caesarean Section

**DOI:** 10.1155/2013/807624

**Published:** 2013-05-27

**Authors:** Madhu Gupta, Shalini Subramanian, Preeti Adlakha

**Affiliations:** Department of Anaesthesiology, ESI-PGIMSR, Basaidara Pur, New Delhi, India

## Abstract

A 31-year-antenatal lady with critical mitral stenosis presented for emergency caesarean section with fetal distress. She had acute onset atrial fibrillation. She was given a combined spinal epidural (CSE) anaesthesia and her arrhythmia was successfully managed after delivery of the baby with intravenous calcium channel blocker. Mitral stenosis is the most common valvular heart disease complicating pregnancy in developing countries. The physiological changes during pregnancy may exacerbate their cardiac symptoms. They may present with complications like congestive cardiac failure, atrial fibrillation, or pulmonary thromboembolism during the antenatal, intrapartum, or postpartum period. Here we discuss the management of parturient woman with high maternal and fetal risk presenting for emergency caesarean. The merits of regional anaesthesia and the importance of invasive monitoring are also discussed.

## 1. Introduction

A 31-year-old lady presented to the antenatal clinic at 34 weeks of gestation with increasing shortness of breath. She was a known case of rheumatic heart disease with mitral stenosis and had undergone balloon mitral valvotomy 12 years ago and closed mitral commissurotomy 7 years ago. She was gravida 8, para 2 with 5 spontaneous abortions and had undergone caesarean section twice since the commissurotomy but had only one living issue who was 3 years old. The other had died a neonatal death. She was on oral Digoxin 0.25 mg od and penicillin prophylaxis since the past seven years. During the present pregnancy, her dyspnea had progressed from NYHA class II to class III. She was put on bed rest and started on diuretics. As part of her workup for elective caesarean section for obstetric reasons, she presented for preanaesthesia evaluation. On auscultation of the heart, she had a mid-diastolic murmur in the mitral area and loud P2. She had no signs of congestive cardiac failure. Her electrocardiogram showed a normal sinus rhythm with a heart rate of 80/min. She had a normal coagulation profile with prothrombin time 13/13, activated partial thromboplastin time 29/31, and platelet count 210 × 10^9^/litre. Her haemoglobin was 11.7 g%. She was advised a fresh echocardiograph and the risk of anaesthesia was explained to her. The next day she presented for emergency CS with onset of preterm labour and a nonreassuring fetal heart rate. She was immediately taken to the operating room. On examination, the patient was dyspneic at rest and unable to lie supine. She had pedal edema and her jugular venous pulse was raised. A 2D echocardiography done only that morning revealed critical mitral stenosis with mitral valve area of 0.7 cm^2^, severe tricuspid regurgitation, mild aortic regurgitation, and pulmonary artery hypertension. Her ejection fraction was 60%. She was propped up with 2 pillows and given O_2_ by face mask at 12 L/min. An intravenous access was secured with an 18G cannula, Ringer lactate was started, and a defibrillator was kept ready. ECG, NIBP, and SpO_2_ monitors were attached. Her pulse was irregularly irregular and the ECG showed atrial fibrillation with a ventricular rate of 142/min. Her blood pressure (BP) was 106/60 mmHg. Her respiratory rate was 32/min but her chest was clear and saturation was 100% with oxygen therapy. A 20G arterial cannula was inserted in the radial artery for invasive blood pressure monitoring. The arterial blood gas analysis showed pH 7.5, PO_2_ 98 mmHg, PCO_2_ 30 mmHg, HCO_3_ 20 mmol/L, Na^+^ 134 mmol/L, and K^+^ 3.8 mmol/L. Combined spinal epidural (CSE) anesthesia was planned for the surgery. An 18G CSE was inserted in the first attempt at L3/4 intervertebral space using loss of resistance to air in the sitting position. In the subarachnoid block, 6 mg (1.2 mL) of 0.5% Bupivacaine with 25 *μ*g fentanyl was injected through a 27G needle after CSF aspiration. The epidural catheter was inserted 5 cm into the epidural space and fixed. The patient was made to lie over 2 pillows as she could not tolerate a supine position. A wedge was also placed below her right hip to allow left uterine displacement to prevent aortocaval compression. Her BP dropped to 72/50 mmHg which increased to 92/54 mmHg with 6 mg ephedrine. The sensory level achieved was T10 and epidural top up of 3 mL of 2% Xylocaine with adrenaline was given in aliquots of 1 mL to achieve a level of T6 when the surgery commenced. She required one more dose of 6 mg Ephedrine to maintain her systolic BP above 90 mmHg. Her ventricular rate rose to 172/min during this period. The baby was delivered within 5 minutes of abdominal incision. The 2.2 kg male baby had aspirated thick meconium and required endotracheal intubation, tracheobronchial suctioning, and mechanical ventilation. After delivery of the baby, 10 units of oxytocin was added to the mother's drip. Her BP was 92/60 mmHg and keeping all preparations ready for emergency cardioversion, she was given 15 mg of Diltiazem injection in 5 mg boluses to obtain a ventricular rate of 90/min. Her ECG reverted to sinus rhythm. Her systolic BP rose to 110 mmHg and she did not require any further vasopressors. A peripherally inserted central venous catheter was inserted to measure the central venous pressure. The rest of the surgery was uneventful. She received 500 mL of RL including 10 units of oxytocin and her urine output was 50 mL. She was shifted to the ICU for postoperative observation and monitoring. In the ICU, she received oxygen therapy, diuretics, epidural analgesia, and intravenous fluids guided by central venous pressure. Invasive BP monitoring was continued for 24 hours. An infusion of amiodarone was used to maintain sinus rhythm and rate. Postoperatively, continuous hemodynamic monitoring was done with invasive monitors, intra-arterial blood pressure, and central venous pressure monitoring. She remained hemodynamically stable and had no episode of tachyarrhythmia, pulmonary edema, or thromboembolism during her stay. The next day, she was started on oral diltiazem and subcutaneous low molecular weight heparin and shifted to the ward on the postoperative day 2 from where she was discharged 7 days later. The neonate developed meconium aspiration pneumonia and died on day 3 of life.

## 2. Discussion

Rheumatic mitral stenosis is not uncommon in developing countries and can complicate up to 88% of pregnant patients with heart disease [1]. As in our case, many of them present for the first time in the third trimester when cardiac symptoms worsen because of the physiological changes of increased cardiac output, heart rate, oxygen consumption, and hemodilution [2]. The preferred mode of delivery in these patients is the vaginal route with labor analgesia to alleviate the increased demand on the heart. Caesarean section is reserved for obstetric indications. The maternal complications that may occur are pulmonary edema, tachyarrhythmia, stroke, cardiac arrest, or death [3–5]. 

Here, we discuss the case of a 34, week antenatal lady with critical mitral stenosis who presented for emergency Caesarean section with acute atrial fibrillation (AF). The acute onset (<48 hours) of her arrhythmia was evident as her preanaesthetic evaluation done the day earlier showed her to be in sinus rhythm. As her BP was 100/60 with severe fetal distress and no symptoms of angina or acute heart failure [6], we decided to urgently deliver the baby and then manage the tachyarrhythmia. Administering general anaesthesia to this patient involved the risk of hemodynamic instability with the use intravenous induction agents. Etomidate [7] has been used but was not available in our hospital. Using high dose opioids was not also an option in view of the parturient being full stomach. Secondly, the stress of laryngoscopy and intubation might precipitate pulmonary edema. Thirdly, cardiovascular collapse or arrest during induction would necessitate urgent hysterotomy and delivery of the baby to facilitate maternal resuscitation [8]. This would increase the maternal and fetal morbidity manifold. Positive pressure ventilation would further reduce the preload. Considering the above risks, the absence of coagulopathy, and the option of providing postoperative epidural analgesia, a regional anaesthesia technique was decided upon. 

The CSE anaesthesia has been used for CS successfully in several cardiac patients [9]. A CSE was given as using spinal anaesthesia alone would cause sudden severe hypotension. The CSE offered the combined advantage of immediate dense blockade, with a low dose spinal, to start the urgent surgical procedure, and the gradual increase in the height of the block with epidural if necessary. The height achieved with the subarachnoid block was only T10. The reason may be small dose of local anaesthetic used and the head up position that the patient had to assume because of her dyspnea. Epidural top up was required to achieve a level of  T6. The hypotension after spinal was successfully managed with Ephedrine injection. 

Once the baby was delivered, the increased venous return enabled us to achieve rate control with Diltiazem. As rate control is not inferior to rhythm control [6] in prevention of death or morbidity, we used calcium channel blocker to achieve rate control. The same drug also provided rhythm control in our patient though this is unusual [6]. Amiodarone infusion was started in the ICU to maintain the sinus rhythm and heart rate <110/min [10]. She required no further vasopressor therapy during the surgery or in the postoperative period. Thromboprophylaxis was started once the risk of surgical bleeding was absent [10]. Our patient had high maternal and fetal risk [5]. The factors predicting primary cardiac events in our patient were NYHA class >II at presentation and left heart obstruction (mitral valve area < 2 cm^2^) [11]. The causes for the poor neonatal outcome [4, 11] were prematurity, meconium aspiration, uterine hypoperfusion, and preexisting intrauterine factors as suggested by her bad obstetric history. In retrospect, our decision to deliver the baby first in the face of maternal tachyarrhythmia was justified as fetal distress due to meconium aspiration would not have responded to merely maternal stabilization and consequent improvement in uterine perfusion. There have been reports in the literature of patients with uncontrolled atrial fibrillation who have been managed and then had an uncomplicated vaginal delivery at a later date [12, 13], but none with acute atrial fibrillation for caesarean section. Acute atrial fibrillation in valvular heart disease has a high risk of heart failure, stroke, and death [6]. The factors that abetted a favourable maternal outcome in our case were good left ventricular ejection fraction (>40%), hemodynamic stability in the face of acute atrial fibrillation, and the use of regional anaesthesia to aid urgent delivery of the gravid uterus, which improved preload and facilitated rhythm control. 
